# On the Determination of Elastic Properties of Single-Walled Boron Nitride Nanotubes by Numerical Simulation

**DOI:** 10.3390/ma14123183

**Published:** 2021-06-09

**Authors:** Nataliya A. Sakharova, Jorge M. Antunes, André F. G. Pereira, Bruno M. Chaparro, José V. Fernandes

**Affiliations:** 1 Centre for Mechanical Engineering, Materials and Processes (CEMMPRE), Department of Mechanical Engineering, University of Coimbra, Rua Luís Reis Santos, Pinhal de Marrocos, 3030-788 Coimbra, Portugal; jorge.antunes@dem.uc.pt (J.M.A.); andre.pereira@dem.uc.pt (A.F.G.P.); bruno.chaparro@ipt.pt (B.M.C.); valdemar.fernandes@dem.uc.pt (J.V.F.); 2 Polytechnic Institute of Tomar, Quinta do Contador, Estrada da Serra, 2300-313 Tomar, Portugal

**Keywords:** boron nitride nanotubes, rigidities, elastic moduli, modelling, numerical simulation

## Abstract

The elastic properties of chiral and non-chiral single-walled boron nitride nanotubes in a wide range of their chiral indices and diameters were studied. With this aim, a three-dimensional finite element model was used to assess their rigidities and, subsequently, elastic moduli and Poisson’s ratio. An extensive study was performed to understand the impact of the input parameters on the results obtained by numerical simulation. For comparison, the elastic properties of single-walled boron nitride nanotubes are shown together with those obtained for single-walled carbon nanotubes.

## 1. Introduction

For the past three decades, carbon nanotubes (CNTs) have been the most widely studied nanostructures due to their well-developed synthesis technologies, remarkable mechanical and physical properties, which provide their numerous potential applications. From a structural point of view, CNTs are cylinders obtained from a honeycomb lattice representing a single atomic layer of crystalline graphite [1]. The honeycomb structures are not restricted to carbon and can be formed by other chemical elements and transition-metal compounds [2,3]. For example, elements of III group of the periodic table (such as boron, aluminium and gallium) are able to establish a strong covalent bond with nitrogen, which leads to a honeycomb arrangement with alternating atoms of the III group element and nitride forming the graphene-like hexagonal lattice [4]. Among these structures, hexagonal boron-nitride (hBN), called white graphene, stands out for its high strength and thermal conductivity, transparency for visible light, antimicrobial properties, relative chemical inertia compared to CNTs, electric insulator characteristics regardless of chirality [5], and also its biocompatibility [6] and high resistance to oxidation [7]. All these characteristics make hBN indispensable for neutron-absorbing materials, for protection of equipment working in a hazardous environment, for many applications in biomedicine, high-temperature catalysts, and nanoscale electronic and photovoltaic devices, as a water purifier and as sensors and bio-detectors [8,9].

The existence of the boron nitride nanotubes (BNNTs) was first predicted theoretically in 1994 [10], and then BNNTs were successfully synthesized in the following year [11]. Having some mechanical and physical properties similar to those of CNTs, BNNTs can replace carbon counterparts in several applications such as sensors [12], hydrogen storage [13], water purification [14] and reinforcement of composites [15]. The structural similarity of CNTs and BNNTs makes it possible to create new hybrid nanostructures, where constituent layers are carbon and non-carbon nanotubes. This procedure allows combining the advantages of each component and obtaining heterostructures with improved properties for innovative applications. For example, a new nanostructure composed of two layers of carbon and boron nitride nanotubes (NTs) has the prospective of replacing double-walled CNTs for high strain applications [16] and has a potential application as the smallest co-axial cable. The latter has been evidenced by the recent accomplishment of the growing of CNT inside the BNNT [17].

In order to take advantage of most of the properties of boron nitride nanotubes for the correct design of their applications, such as BNNTs-reinforced composites and hybrid structures of BNNTs with CNTs, the knowledge of their mechanical properties is of utmost importance. Firstly, because the performance and robustness of nanosystems and nanodevices containing BNNTs depend on their mechanical behaviour, and also because the deformation can influence physical properties, such as optical, electric, thermoelectric and chemical, of boron nitride NTs [8,18,19].

Similar to the studies focused on the mechanical characterization of CNTs, those regarding the BNNTs’ mechanical behaviour are predominantly carried out resorting to theoretical (analytical and numerical) methods due to the high cost and high resource of experimental procedures at the nanoscale. As with carbon nanotubes, three classes of the theoretical approaches have been used to model and characterize the mechanical behaviour of BNNTs, namely, the atomistic approach, which comprises ab initio [20] and molecular dynamics (MD) [3,21,22,23,24,25,26], the continuum mechanics (CM) approach [27,28] and the nanoscale continuum modelling (NCM) approach, also called molecular structural mechanics (MSM) [29,30,31,32,33,34,35,36]. Among the works in which atomistic modelling was used, the elastic properties of BNNTs were accessed with recourse to MD simulations using different analytical or empirical potential functions for describing the interactions between boron (B) and nitride (N) atoms in the nanotubes. Choyal et al. [23] performed MD simulations with Tersoff–Brenner (TB) potential to study the influence of the aspect ratio on the BNNTs’ Young’s modulus. Verma et al. [21] used the TB potential with modified parameters to calculate the bending energy and, consequently, the Young’s and shear moduli and the Poisson’s ratio of BNNTs with different diameters. Tao et al. [24] integrated MD simulation with TB potential and finite element (FE) method to access Young’s modulus and study the buckling behaviour of BNNTs. In their MD simulation study, Vijayaraghavan and Zhang [22] adopted REBO, a second-generation reactive empirical bond order, to describe the interactions between B and N atoms and modelled the BNNTs’ mechanical behaviour under tensile loading. Santosh et al. [25] used MD simulation, with a force-constant approach to depict the interaction between B and N atoms under axial compression to study buckling behaviour of BNNTs and calculate their Young’s and shear moduli. About other atomistic approaches, Kochaev [20] used ab initio simulation to evaluate Young’s modulus and Poisson’s ratio of BNNTs, while Hernandez et al. [3] evaluated these elastic properties using tight-binding molecular dynamics (TBMD). Zhang et al. [26] coupled MD computational approach with the density-functional-based tight-binding (DFTB) model to evaluate the Young’s and shear moduli of BNNTs.

With respect to the CM approach, which consists of modelling the nanotube as a continuum structure, Oh [27] employed analytical continuum lattice (CL) thermodynamic approach, combined with the adjusted TB potential, to describe the interaction between B and N atoms and to evaluate Young’s modulus and Poisson’s ratio of BNNTs. Song et al. [28] proposed a finite-deformation shell model to study the instabilities of BNNTs under different loading conditions in tension, compression and torsion. Uzun et al. [37] modelled whole BNNT structures as nano-scaled beams using Social Spider Optimization (SSO) algorithm to design the beams with optimum cross-sectional areas. In their work [37], the displacements of BNNT (beam) were analysed with recourse of Euler–Bernoulli beam theory, the same employed by Ouakad et al. [38] to study the fundamental frequencies of hybrid boron-nitride-carbon nanotubes.

In the NCM/MSM approach, which considers the connection between the molecular configuration of nanotube and the solid mechanics, BNNT is comprehended as a space frame structure, where the covalent bonds between B and N atoms are simulated with elements (such as beams or springs) as in elasticity theory. Salavati et al. [30], Li and Chou [31] and Ansari et al. [33] used the NCM/MSM approach, in which the B–N bond is replaced by the beam element, to study the buckling behaviour [33], elastic moduli and dynamic properties [31] and electromechanical properties [30] of BNNTs. Moreover, in another study, Ansari et al. [34] used closed-formed analytical solutions based on a molecular mechanics model to assess the surface Young’s modulus and Poisson’s ratio of BNNTs. In the works of Jiang and Guo [32] and Genoese et al. [35], based on the NCM/MSM approach, an analytical “stick-and-spring” model for single-walled BNNTs was used to evaluate their surface elastic moduli and Poisson’s ratio. Additionally, within the framework of NCM/MSM approach, Yan and Liew [29] considered a representative cell built by B atom connected to three neighbouring N atoms by B–N covalent bonds to construct the BNNT model, and then, the structural mechanic parameters were determined by minimizing the potential of the representative cell. Yan et al. [36] studied longitudinal and torsional free vibrations of BNNTs under the NCM/MSM approach coupled with the Euler beam model and obtained analytical solutions for fundamental frequencies and shear modulus.

The crucial point in the application of the NCM/MSM approach is to deduce the elastic properties of elements that simulate covalent B–N bond, using a linkage between the parameters of the structural mechanic of the elastic element and the molecular mechanics parameters, namely force-field constants. If in the case of modelling of CNTs, the choice of the force-field constants, which provide input for numerical or analytical models, was unambiguous, computation of these constants for the case of BNNTs has become a challenge for the research community. Different calculation methods have been already used to assess the force constants for BNNTs, and, as a result, a scattering of BNNTs elastic properties reported in the literature is evident.

The aim of the present study is to characterize the mechanical behaviour of single-walled boron nitride nanotubes (SWBNNTs) for a broad range of chiral indices, diameter and length, employing the NCM/MSM approach with beam elements. The three-dimensional (3D) FE method was employed to proceed with a systematic evaluation of the bending, torsional and tensile rigidities, and, subsequently, the shear and Young’s moduli, and the Poisson’s ratio of SWBNNTs. A comprehensive study of the influence of input parameters chosen for the FE modelling on the computed elastic properties of BNNTs was carried out. Since boron nitride nanotubes have a great potential to substitute their carbon counterparts in practical applications and taking into account the increased prospective of hybrid nanostructures, consisting of boron nitride and carbon nanotubes, the results thus obtained were compared with those for single-walled carbon nanotubes (SWCNTs). Furthermore, the present study provides a benchmark with regard to determining the mechanical properties of SWBNNTs and a guide for the correct design of hybrid SWBNNT/SWCNT structures.

## 2. Materials and Methods

### 2.1. Atomic Structure of SWBNNTs

**Figure 1:**
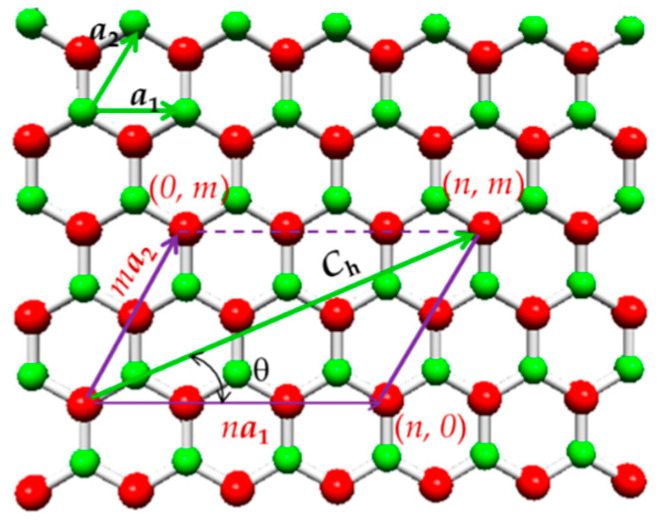
The scheme of an unrolled hexagonal boron nitride sheet with the definition of the chiral vector, **C_h_**, and the chiral angle, θ.

As in the case of an SWCNT, an SWBNNT can be understood as a rolled-up hexagonal boron nitride sheet, and the atomic structure of the nanotube is characterized by the chirality that is expressed by the chiral vector, **C_h_**, and the chiral angle, θ, as shown in Figure 1.

The chiral vector is given as follows:

(1) $$C_{h}=n\cdot a_{1\;}+m\cdot a_{2}$$

where $a_{1}$ and $a_{2}$ are the unit vectors of the hexagonal BN lattice, and n and m are chiral indices (always integers). The length of the unit vector a is defined as $a=\sqrt{3}\cdot a_{B-N}$, where $a_{B–N}$ is the equilibrium B–N covalent bond length. Unlike the carbon–carbon (C–C) bond length, $a_{C–C}$, which value is usually considered equal to 0.1421 nm [1]. Various B–N bond length values have been reported in the literature (see Table 1).

The nanotube circumference, L_c_, and the diameter, $D_{n}$, are expressed as follows:

(2) $$L_{c}=\left|C_{h}\right|=a\cdot \sqrt{n^{2}+n\cdot {m+m}^{2}}$$

(3) $$D_{n}=\frac{L_{c}}{\pi }=\frac{a_{B-N}\cdot \sqrt{3\cdot \left(n^{2}+n\cdot {m+m}^{2}\right)}}{\pi }$$

The chiral angle, θ, is defined by the angle between the chiral vector, **C_h_**, and the direction (n, 0), and is given by:

(4) $${\theta =\sin}^{-1}\frac{\sqrt{3\cdot }m}{2\sqrt{n^{2}+n\cdot {m+m}^{2}}}$$

**Figure 2:**
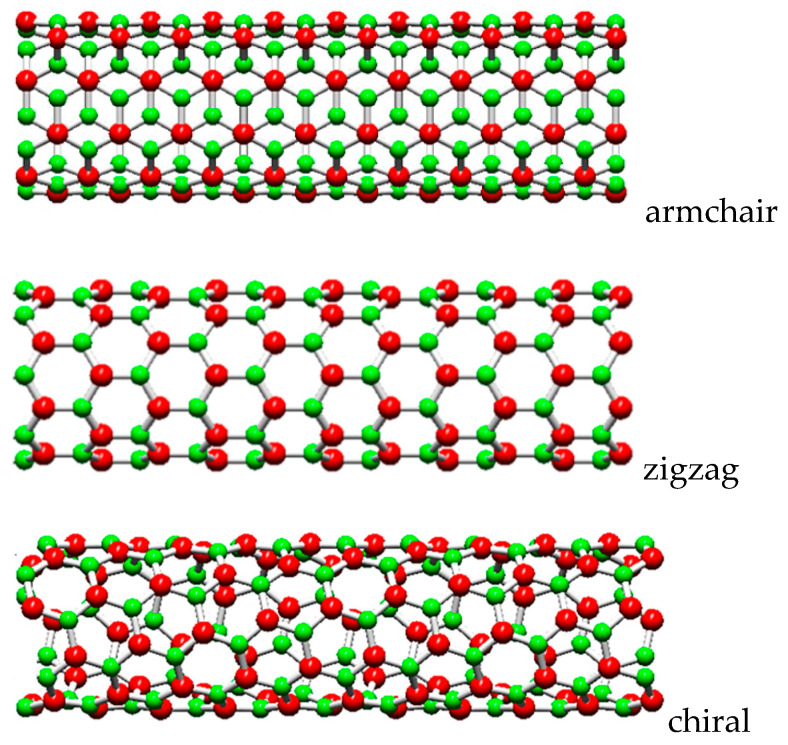
The main symmetry groups of BNNTs acquired using the software Nanotube Modeler© (version 1.8.0, ©JCrystalSoft).

As for the SWCNTs, the chiral angles for the SWBNNTs are in the range between 0° and 30°, defining three main symmetry groups: non-chiral nanotubes for the 2 limiting cases of θ = 0° (zigzag) and θ = 30° (armchair), and chiral nanotubes for 0° < θ < 30°. In terms of chiral indices, when n = m, the structure corresponds to the armchair configuration (n, n); when m = 0, the structure corresponds to the zigzag configuration (n, 0); when n ≠ m, the structure is chiral (n, m). Schematic representations of armchair, zigzag and chiral SWBNNTs are shown in Figure 2.

### 2.2. Molecular Structure of SWBNNTs and Equivalent Properties of Elastic Beams

The NCM/MSM approach employed in the present study to determine the elastic properties of SWBNNTs is based on the connection between the inter-atomic potential energies associated with bond interactions in the molecular system and the strain energies of the equivalent continuum structure, composed by beam elements undergoing axial, bending and torsional deformations. In this way, the elastic properties of the beams are determined using molecular mechanics (MM) relationships, as was established by Li and Chou [42] for CNTs.

Based on molecular mechanics, the total potential energy of a molecular system is given as the sum of the energy terms, owing to bonded and non-bonded interactions [43,44]:

(5) $$U_{\mathrm{tot}}{=U}_{r}{+U}_{\theta }{+U}_{\phi }{+U}_{\omega }{+U}_{\mathrm{nb}}$$

where U_r_, U_θ_, U_φ_ and U_ω_ are energies related to bond stretching, bond bending, dihedral angle torsion and out-of-plane torsion, respectively, and U_nb_ is the energy related to non-bonded interactions that consist of van der Waals, electrostatic and explicit hydrogen bonds terms. In covalent systems, such as boron nanotubes, non-bonded interactions are negligible when compared with bonded ones [31] and the principal contribution to the total potential energy comes from the first four terms of Equation (5). Considering that the potential energies are adequately described by the harmonic approximation under the assumption of small deformation, and merging the dihedral angle torsion and out-of-plane torsion into a single equivalent term, U_τ_ = U_φ_ + U_ω_, Equation (5) can be rewritten as follows:

(6) $$U_{\mathrm{tot}}{=U}_{r}{+U}_{\theta }{+U}_{\tau }=\frac{1}{2}\cdot k_{r}\cdot {\left(\Delta r\right)}^{2}+\frac{1}{2}\cdot k_{\theta }\cdot {\left(\Delta \theta \right)}^{2}+\frac{1}{2}\cdot k_{\tau }\cdot {\left(\Delta \phi \right)}^{2}$$

where k_r_, k_θ_ and k_τ_ are the bond stretching, bond bending and torsional resistance force constants, respectively, and Δr, Δθ and Δφ are the bond stretching increment, bond angle bending variation and angle variation of twist bond, respectively.

Regarding the bond force constants for the BN nanostructures, different values of k_r_, k_θ_ and k_τ_, depending on the methods for their calculation, have been reported in the literature, as resumed in Table 2. Among the well-known generic molecular force fields, only UFF (Universal Force Fields) [43] and DREIDING force field [44] have the necessary parameters to describe the B–N bonds and, consequently, directly calculating the bond force constants. As it was shown by Rappé et al. [43] (UFF), the bond-bending constant, k_θ_, of the diatomic nanostructure, which is the case of that constituted by the B and N atoms, depends on the lengths of B–N and N–B bonds, the three-body angles between pairs of bonds B–N–B and N–B–N and the effective charges of the atoms B and N. As a result, there are two different values for the bond-bending constant, k_θ__1_ and k_θ__2_, related with effective charges of the atoms ($Z_{1}^{*2}$) by following expression [43]:

(7) $$\frac{k_{\theta 1}}{k_{\theta 2}}=\frac{Z_{2}^{*2}}{Z_{1}^{*2}}$$

In the DREIDING force field, which is well-defined for any pair of atoms, it is not necessary to distinguish two different bond-bending constants. For this reason, DREIDING was adopted by Li and Chou [31], however, with a modification for the bond torsion. The other method for determining force constants is the use of the density functional theory (DFT). To calculate the bond force constants, Jiang and Guo [32] and Genoese et al. [45] used results available in the literature on ab initio DFT, replacing them in the analytical expressions for surface elastic moduli and Poisson’s ratio, derived from MM models. Ansari et al. [33] computed the force constants in a similar way; however, the surface Young’s modulus, flexural rigidity and Poisson’s ratio to be replaced in the MM relationships were obtained from DFT’s own calculations. Tapia et al. [41] calculated the bond force constants directly, using ab initio DFT computations.

The highest bond stretching constant is calculated by Rappé et al. [43] using the UFF method, k_r_ = 676 nN/nm, and the smallest is predicted considering the DREIDING force field [44], k_r_ = 487 nN/nm. The k_r_ values reported by Ansari et al. [33] and Tapia et al. [41], who employed the DFT-based method for evaluation of the bond stretching constant, are nearly the same and slightly lower than those obtained by Rappé et al. [43] (UFF). The k_r_ force constants calculated in the works of Jiang and Guo [32] and Genoese et al. [45] are close to each other due to the similar calculation approach used and slightly higher than the k_r_ value provided by Mayo et al. [44] (DREIDING).

Regarding the bond-bending constant, k_θ_, an uncertainty in relation to its value is evident when the results available literature is examined. In the studies by Rappé et al. [43] (UFF), Jiang and Guo [32] (DFT) and Genoese et al. [45] (DFT), two values of k_θ_ were calculated taking into consideration the B–N–B and N–B–N configurations. Jiang and Guo [32] and Genoese et al. [45], who shared a similar approach for calculation bond-bending constant, derived comparable pairs of k_θ__1_ and k_θ__2_ values, but different from those provided by Rappé et al. [43]. The value of k_θ_ calculated by Tapia et al. [41] (DFT) is close to that found by Mayo et al. [44] (DREIDING). The bond torsion constant, k_τ_, values reported in the literature for BN nanostructures are even scarcer and with greater scatter than those available for k_r_ and k_θ_ force constants. To our knowledge, apart from the k_τ_ value calculated basing on the DREIDING force field [44] and modification for k_τ_ proposed by Li and Chou [31], only Ansari et al. [33] and Tapia et al. [41] reported in their studies k_τ_ values obtained using the DFT method.

For the stretching, U_A_, bending, U_T_, and torsional, U_M_, energies of a beam under pure axial force, N, pure bending moment, M, and a pure torsion moment, T, respectively, classical mechanics give the following expressions:

(8) $$U_{A}=\frac{1}{2}\cdot \int_{0}^{L}\frac{N^{2}}{E_{b}\cdot A_{b}}dl=\frac{1}{2}\cdot \frac{E_{b}\cdot A_{b}}{l}\cdot {\left(\Delta l\right)}^{2}$$

(9) $$U_{M}=\frac{1}{2}\cdot \int_{0}^{L}\frac{M^{2}}{E_{b}\cdot I_{b}}dl=\frac{1}{2}\cdot \frac{E_{b}\cdot I_{b}}{l}\cdot {\left(2\cdot \tau \right)}^{2}$$

(10) $$U_{T}=\frac{1}{2}\cdot \int_{0}^{L}\frac{T^{2}}{G_{b}\cdot J_{b}}dl=\frac{1}{2}\cdot \frac{G_{b}\cdot J_{b}}{l}\cdot {\left(\Delta \beta \right)}^{2}$$

where *l* is the beam length; A_b_, I_b_ and J_b_ are the cross-section area, the moment of inertia and the polar moment of inertia of the beam, respectively; Δ*l* is the beam axial stretching displacement; τ is the rotational angle at the ends of the beam; Δβ is the relative rotation between the ends of the beam; E_b_ and G_b_ are Young’s and shear moduli of the beam, respectively.

The Equations (6) and (8)–(10) allow establishing the equivalence between molecular and structural systems, i.e., between the stretching energies, U_r_ and U_A_, the bending energies, U_θ_ and U_M_, and the torsional energies, U_τ_ and U_T_. In addition, it can be established the equivalences between the beam axial stretching, Δ*l*, and the bond stretching increment, Δr, the rotational angle, τ, and the total variation of the bond angle, Δθ, and the relative rotation between the beam ends, Δβ, and the twist bond angle variation, Δφ. Thus, the tensile, $E_{b}A_{b}$, bending, $E_{b}I_{b}$, and torsional, $G_{b}J_{b}$, rigidities of beam elements, can be expressed through the force constants k_r_, k_θ_, k_τ_ and the beam length, *l* [42]:

(11) $$E_{b}A_{b}=l\cdot k_{r},\;E_{b}I_{b}=l\cdot k_{\theta },\;G_{b}J_{b}=l\cdot k_{\tau }$$

Equation (11), together with the assumption of equivalence between the beam length, *l,* and the bond length, $a_{B–N}$, are the basis for the analysis of the mechanical behaviour of BNNTs, using continuum mechanics. Assuming a circular cross-section area of the beam element, its cross-section area, A_b_, the moment of inertia, I_b_, and the polar moment of inertia, J_b_, are expressed as follows:

(12) $$A_{b}=\pi \cdot d^{2}/4,\;I_{b}=\pi \cdot d^{4}/64,\;J_{b}=\pi \cdot d^{4}/32$$

where d is the beam diameter.

Combining Equations (11) and (12), the diameter, d, and the Young’s modulus, E_b_, and the shear modulus, G_b_, of the beam to be used as an input in numerical simulation studies can be derived as follows:

(13a) $$d=4\cdot \sqrt{\frac{k_{\theta }}{k_{r}}}$$

(13b) $$E_{b}=\frac{k_{r}^{2}\cdot l}{4\cdot \pi \cdot k_{\theta }}$$

(13c) $$G_{b}=\frac{k_{r}^{2}\cdot k_{\tau }\cdot l}{8\cdot \pi \cdot k_{\theta }^{2}}$$

When two values of bond-bending rigidity, k_θ__1_ and k_θ__2_, are considered the Equations (13a)–(13c) are presented as:

(14a) $$d=2\cdot \sqrt{\frac{2\cdot \left(k_{\theta 1}{+k}_{\theta 2}\right)}{k_{r}}}$$

(14b) $$E_{b}=\frac{k_{r}^{2}\cdot l}{2\cdot \pi \cdot \left(k_{\theta 1}{+k}_{\theta 2}\right)}$$

(14c) $$G_{b}=\frac{k_{r}^{2}\cdot k_{\tau }\cdot l}{2\cdot \pi \cdot {\left(k_{\theta 1}{+k}_{\theta 2}\right)}^{2}}$$

The Poisson’s ratio of the beam element can be calculated by the relationship obtained with recourse to MM models [46] as follows:

(15a) $$\nu_{b\;}=\frac{k_{r}\cdot l^{2}\;–\;6\cdot k_{\theta }}{k_{r}\cdot l^{2}+18\cdot k_{\theta }}$$

which in the case of two different values of k_θ_ is modified as follows [32,45]:

(15b) $$\nu_{b}=\frac{k_{r}\cdot l^{2}\;–\;3\cdot \left(k_{\theta 1}{+k}_{\theta 2}\right)}{k_{r}\cdot l^{2}+9\cdot \left(k_{\theta 1}{+k}_{\theta 2}\right)}$$

The geometrical and mechanical properties of the beam elements for the input of FE models of SWBNNTs and SWCNTs are summarized in Table 3. Five different sets of input parameters for numerical simulation of SWBNNTs were chosen based on the force constants presented in Table 2.

### 2.3. Configurations of Nanotubes and FE Analysis

**Figure A1:**
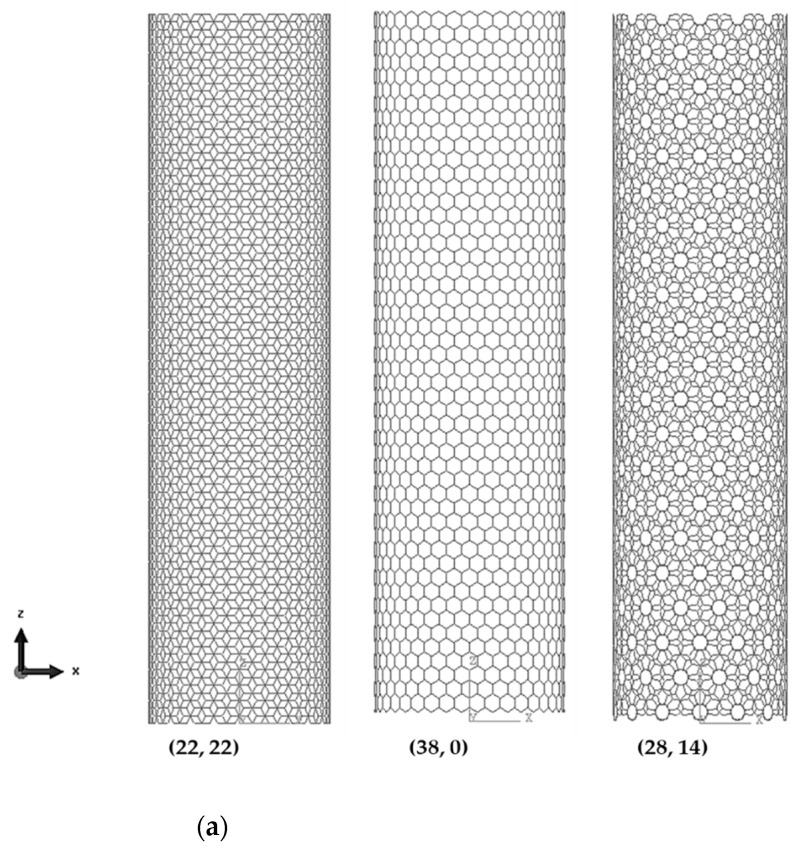

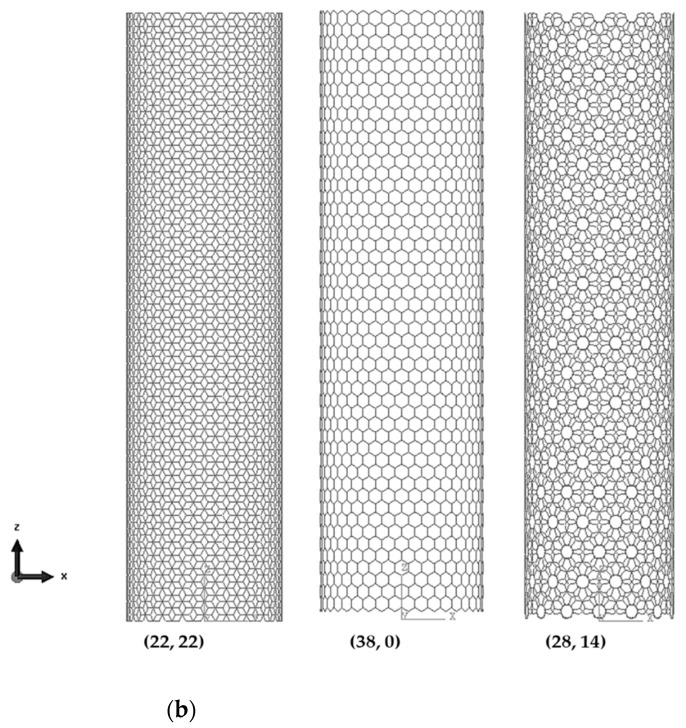
FE meshes of (**a**) SWBNNTs and (**b**) SWCNTs.

The meshes of the SWBNNTs and SWCNTs for FE analyses were built using the Nanotube Modeler© software (version 1.8.0, ©JCrystalSoft), which produces PDB (Program Database) files containing the atom positions and their connections to be used as input data in the commercial FE code ABAQUS^®^ (Abaqus 2020, Dassault Systèmes^®^). To convert the PDB files obtained from the Nanotube Modeler© software to the format compatible with the ABAQUS^®^ code, the in-house application, entitled InterfaceNanotubes.NM*,* was used, which is a modified version of the previously developed InterfaceNanotubes application [50]. Table 4 summarizes the geometric characteristics of the SWBNNTs and SWCNTs used in the current FE analyses. The length of the SWBNNTs and SWCNTs was about 30× greater than the nanotube diameter; in this way, the mechanical behaviour of the nanotube does not depend on the length. Examples of the SWBNNTs and SWCNTs finite element meshes used are shown in Appendix A (Figure A1), and the number of elements and nodes constituting the FE meshes are summarized in Table A1.

The mechanical behaviour of the SWBNNTs and SWCNTs was studied numerically under tensile, bending, and torsion loading conditions, using the FE code ABAQUS^®^. As a result, the EA, EI and GJ, rigidities of the nanotubes are determined as follows:

(16) $$\mathrm{EA}=\frac{F_{a}\cdot L}{u_{a}}$$

(17) $$\mathrm{EI}=\frac{F_{t}\cdot L^{3}}{3\cdot u_{t}}$$

(18) $$\mathrm{GJ}=\frac{T\cdot L}{\varphi }$$

where L is the nanotube length; F_a_, F_t_ and T are the axial tensile force, the transverse force and the torsional moment, respectively, applied at one end of the nanotube, leaving the other fixed; u_a_, u_t_ and ϕ are the axial displacement, the transverse displacement and the twist angle, respectively, obtained from the FE analysis. During the torsion test, the nodes under loading at the end of the nanotube are not permitted to move in the radial direction.

### 2.4. Elastic Constants of SWBNNTs

As in the case of SWCNTs [50,51], the Young’s, E, and shear, G, moduli, and the Poisson’s ratio, ν, of SWBNNTs can be assessed with recourse to the results of tensile, EA, bending, EI, and torsional, GJ, rigidities. The SWBNNTs with the mean diameter $\bar{D}$ and the nanotube wall thickness, $t_{n}$, have the cross-sectional area, A, the moment of inertia, I, and the polar moment of inertia, J, of the equivalent hollow cylinder expressed respectively by:

(19) $$A\;=\;\frac{\pi }{4}\cdot [{\left(\bar{D}+t_{n}\right)}^{2}-{\left(\bar{D}-t_{n}\right)}^{2}]=\pi \bar{D\cdot }t_{n}$$

(20) $$I\;=\;\frac{\pi }{64}\cdot [{\left(\bar{D}+t_{n}\right)}^{4}-{\left(\bar{D}-t_{n}\right)}^{4}]=\frac{\pi {\bar{\cdot D}}^{3}\cdot t_{n}}{8}\cdot [1+{\left(\frac{t_{n}}{\bar{D}}\right)}^{2}]$$

(21) $$J\;=\;\frac{\pi }{32}\cdot [{\left(\bar{D}+t_{n}\right)}^{4}-{\left(\bar{D}-t_{n}\right)}^{4}]=\frac{\pi {\bar{\cdot D}}^{3}\cdot t_{n}}{4}\cdot [1+{\left(\frac{t_{n}}{\bar{D}}\right)}^{2}]$$

The knowledge of the EA and EI rigidities and Equations (19) and (20) allow determining the diameter $\bar{D}$ as follows:

(22) $$\frac{\mathrm{EI}}{\mathrm{EA}}=\frac{1}{8}\cdot \left({\bar{D}}^{2}+t_{n}^{2}\right)\Rightarrow \bar{D}=\sqrt{8\cdot \left(\frac{\mathrm{EI}}{\mathrm{EA}}\right)\cdot t_{n}^{2}}$$

Consequently, substituting in the Equations (19) and (21) the mean diameter, $\bar{D}$, given by Equation (22), the E and G moduli can be calculated using the following expressions, respectively:

(23) $$E=\frac{\mathrm{EA}}{A}=\frac{\mathrm{EA}}{\pi \cdot t_{n}\cdot \sqrt{8\cdot \left(\frac{\mathrm{EI}}{\mathrm{EA}}\right)–t_{n}^{2}}}$$

(24) $$G=\frac{\mathrm{GJ}}{J}=\frac{\mathrm{GJ}}{2\cdot \pi \cdot t_{n}\cdot \left(\frac{\mathrm{EI}}{\mathrm{EA}}\right)\cdot \sqrt{8\cdot \left(\frac{\mathrm{EI}}{\mathrm{EA}}\right)–t_{n}^{2}}}$$

In the current study, the value of the SWBNNTs wall thickness was considered equal to the graphite interlayer spacing, $t_{n}$ = 0.34 nm, as observed experimentally by transmission electron microscopy ($t_{n}$ = 0.338 ± 0.004 nm [52]) and calculated by the theoretical approaches [43,44,45,46,47,48,49,50,51,52,53,54,55]. However, there is no agreement on the value of $t_{n}$ and although the values above mentioned are commonly used, Tapia et al. [41] and Boldrin et al. [56] reported $t_{n}$ = 0.106 nm, and Vijayaraghavan and Zhang [22] calculated $t_{n}$ = 0.105 nm. Additionally, $t_{n}$ values equal to 0.936 nm [57] and 0.33 nm [21,58] can be found in the literature.

Assuming the isotropy condition and taking into account that J = 2·I, the Poisson’s ratio can be calculated from the EI and GJ rigidities, as follows:

(25) $$\nu =\frac{E}{2\cdot G}\;–\;1=\frac{\mathrm{EI}}{\mathrm{GJ}}\;–\;1$$

## 3. Results and Discussion

### 3.1. Rigidities of SWBNNTs: Parametric Studies on the Effect of Diameter, Chiral Angle and Aspect Ratio

**Figure 3:**
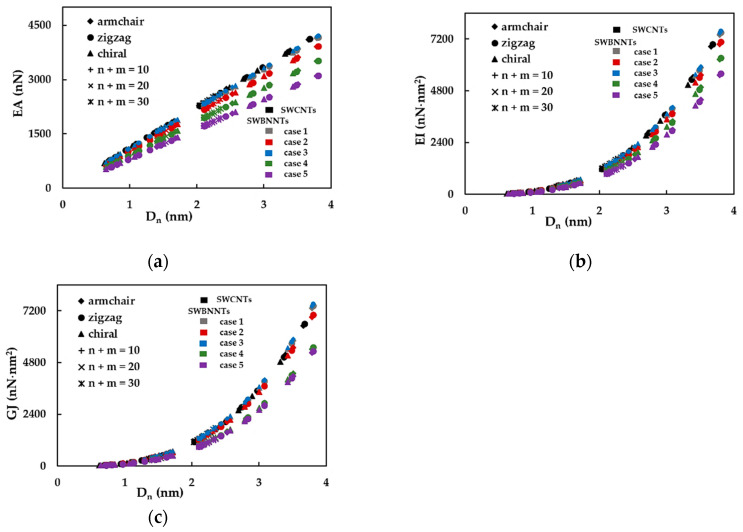
The evolution of rigidity as a function of the nanotube diameter, $D_{n}$, for non-chiral and chiral SWBNNTs considering five different cases of input parameters and for SWCNTs with the same chiral indices in: (**a**) tension, EA, (**b**) bending, EI, and (**c**) torsion, GJ. See also Table 3.

**Figure 4:**
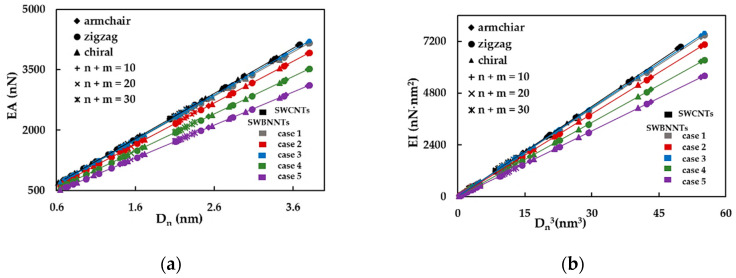

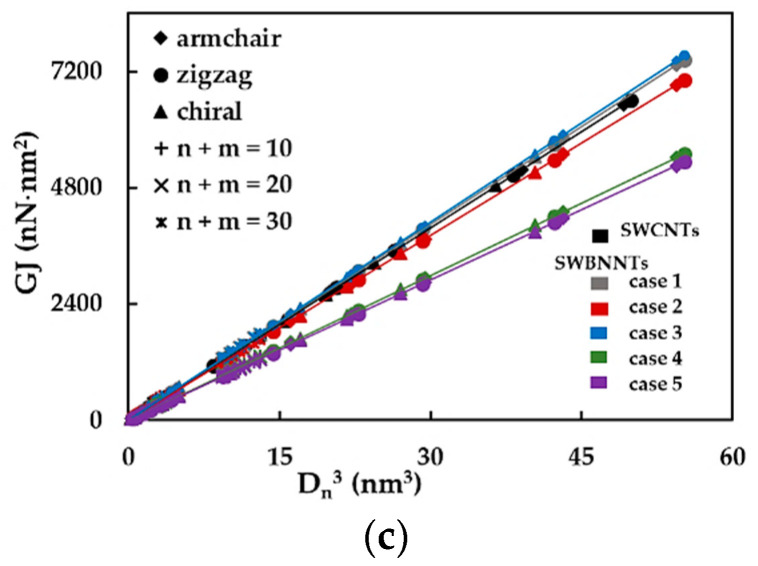
The evolution of rigidities as a function of the nanotube diameter, $D_{n}$, for non-chiral and chiral SWBNNTs, considering five different cases of input parameters, and for SWCNTs with the same chiral indices in: (**a**) tension, EA, (**b**) bending, EI, and (**c**) torsion, GJ.

First, the effect of the nanotube diameter on the SWBNNTs rigidity was studied using numerical simulations as above described. The tensile, bending and torsional rigidities of the SWBNNTs, obtained by Equations (16)–(18), for the five cases of numerical simulation input values shown in Table 3, are presented as a function of the nanotube diameter, $D_{n}$, in Figure 3a–c, respectively. The rigidity results for SWCNTs are also plotted in Figure 3a–c for comparative purposes. For each case of numerical simulation input values shown in Table 3, the rigidity results appear along the same alignment, regardless of the type of nanotube, chiral or non-chiral, and the corresponding diameter; the same is true of the results of the SWCNTs, as it was already observed [50,51]. The evolutions of the EA rigidity obtained for cases 1 and 3 of the input parameters for SWBNNTs almost coincide with each other and with the EA evolution obtained for SWCNTs. The EA values for cases 2, 4 and 5 are lower than for cases 1 and 3 and decrease from case 2 to case 5. The same is true for the evolutions of the EI rigidity. The evolutions of GJ rigidity for cases 1 and 3 of SWBNNTs and for SWCNTs are nearly coincident, and the same is valid for cases 4 and 5. The GJ values for case 2 are lower than in cases 1 and 3 and higher than in cases 4 and 5. Based on the expressions (19), for cross-section area and (20) and (21), for moments of inertia of nanotube, the values of the tensile rigidity, EA, shown in the Figure 3a are represented as a function of nanotube diameter, $D_{n}$, and the values of the bending, EI, and torsional, GJ, rigidities as shown in Figure 3b,c, respectively, are plotted as a function of $D_{n}^{3}$, in Figure 4a–c.

As previously found for the case of the SWCNTs [50,51], the following expressions describing the straight lines in the Figure 4a–c can be written:

(26) $${\mathrm{EA}}_{\mathrm{BN}}{=\alpha }_{\mathrm{BN}}\cdot D_{n}$$

(27) $${\mathrm{EI}}_{\mathrm{BN}}{=\beta }_{\mathrm{BN}}\cdot D_{n}{}^{3}$$

(28) $${\mathrm{GJ}}_{\mathrm{BN}}{=\gamma }_{\mathrm{BN}}\cdot D_{n}{}^{3}$$

where $\alpha_{\mathrm{BN}}$, $\beta_{\mathrm{BN}}$ and $\gamma_{\mathrm{BN}}$ are the fitting parameters. The values of the $\alpha_{\mathrm{BN}}$, $\beta_{\mathrm{BN}}$ and $\gamma_{\mathrm{BN}}$ obtained from Figure 4a–c for SWBNNTs are resumed in Table 5.

Equations (26)–(28) are similar to those obtained in the authors preceding works for the case of the SWCNTs: ${\mathrm{EA}}_{C}{=\alpha }_{C}\cdot \left(D_{n}{–\;D}_{0}\right)$, ${\mathrm{EI}}_{C}{=\beta }_{c}\cdot {\left(D_{n}–D_{0}\right)}^{3}$, ${\mathrm{GJ}}_{C}{=\gamma }_{c}\cdot {\left(D_{n}{–\;D}_{0}\right)}^{3}$ with the fitting parameters $\alpha_{C}$, $\beta_{C}$, $\gamma_{C}$ and $D_{0}$ [50,51]. For SWCNTs the fitting parameters estimated based on the results of Figure 4a–c are: $\alpha_{C}\;$= 1119.89 nN/nm, $\beta_{C}$ = 139.40 nN/nm and $\gamma_{C}$ = 132.44 nN/nm, which is close to those previously calculated: $\alpha_{C}\;$= 1121.20 nN/nm, $\beta_{C}$ = 140.25 nN/nm, $\gamma_{C}$ = 130.39 nN/nm and $D_{0}$ was considered equal to zero, since its value is negligible when compared with the nanotube diameter $D_{n}$ [59].

The mean differences between the values of EA, EI and GJ rigidities calculated with Equations (26)–(28), respectively, and the corresponding rigidity values obtained directly from FE analysis are shown in Table 6. These results allow us to conclude that Equations (26)–(28) make an accurate assessment of the tensile, bending and torsional rigidities of SWBNNTs. The greatest mean difference, which is observed for the EI values, is less than 1%.

**Figure 5:**
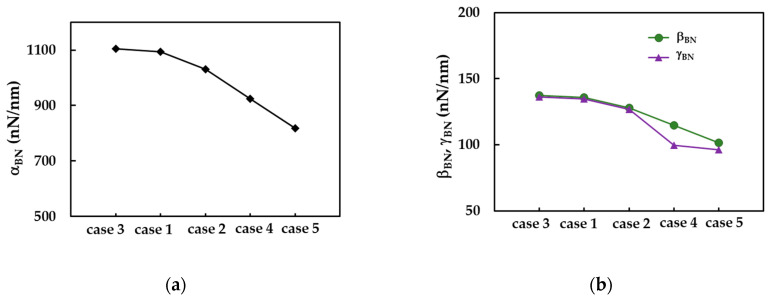
The evolutions of: (**a**) $\alpha_{\mathrm{BN}}$, and (**b**) $\beta_{\mathrm{BN}}$ and $\gamma_{\mathrm{BN}}$ fitting parameters for the five cases of input values in the numerical simulation of SWBNNTs.

The evolutions of $\alpha_{\mathrm{BN}}$, $\beta_{\mathrm{BN}}$ and $\gamma_{\mathrm{BN}}$ for five cases of input parameters, ordered by decreasing values of the rigidities, are shown in Figure 5. It should be noted that for cases 1, 2 and 3, the ratio $\beta_{\mathrm{BN}}$/$\gamma_{\mathrm{BN}}$ is about 1.01, and for case 5, $\beta_{\mathrm{BN}}$/$\gamma_{\mathrm{BN}}$ = 1.05, which means that the values of bending, EI, and torsional, GJ, rigidities are close to each other. For case 4, the value of the ratio $\beta_{\mathrm{BN}}$/$\gamma_{\mathrm{BN}}$ is 1.15, which corresponds to the highest ratio between the values of the EI and GJ rigidities, observed among the studied five cases of input values in numerical simulations.

**Figure 6:**
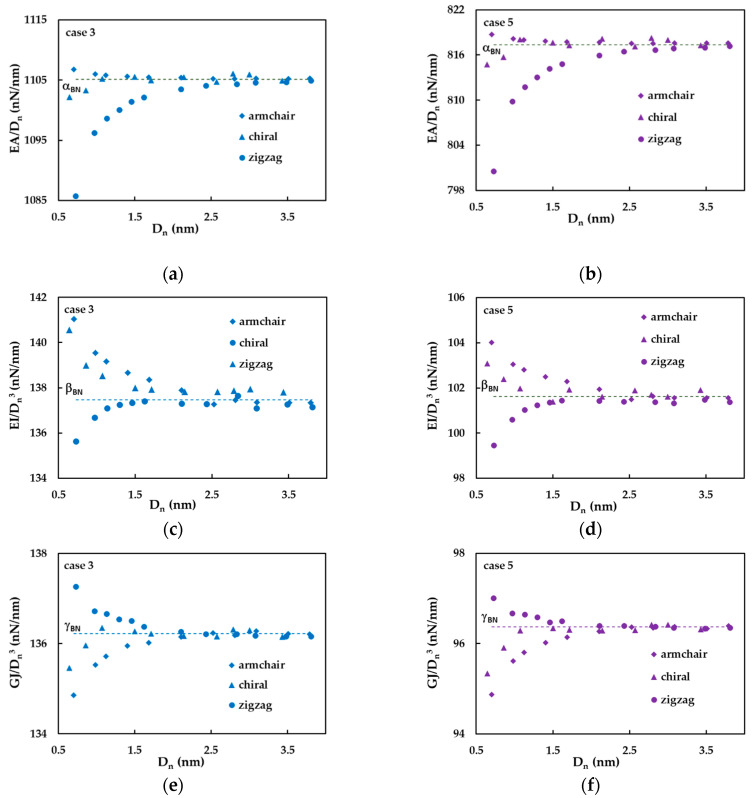
The evolutions of the ratios EA/$D_{n}$ (**a**,**b**), EI/$D_{n}^{3}$ (**c**,**d**) and GJ/$D_{n}^{3}$ (**e**,**f**) with the SWBNNT diameter, $D_{n}$, for case 3 (**a**,**c**,**e**) and case 5 (**b**,**d**,**f**).

A careful analysis of the numerical results shows that the Equations (26)–(28) for the determination of the tensile, EA, bending, EI and torsional, GJ, rigidities, respectively, do not fit the three rigidities of SWBNNTs with enough accuracy, in all range of SWBNNTs diameters. For better understanding, the ratios EA/$D_{n}$, EI/$D_{n}^{3}$ and GJ/$D_{n}^{3}$ were represented as a function of nanotube diameter, $D_{n}$, in Figure 6a–f, respectively. Two cases of the input parameters, cases 3 and 5, were considered for this analysis.

The ratios EA/$D_{n}$ and EI/$D_{n}^{3}$ are almost constant and equal to the values of the fitting parameters $\alpha_{\mathrm{BN}}$ (Equation (26)) and $\beta_{\mathrm{BN}}$ (Equation (27)), respectively, for the SWBNNTs with diameters $D_{n}$ > 1.5 nm. For SWBNNTs with $D_{n}$ values less than 1.5 nm, the EA/$D_{n}$ ratio slightly increases for armchair (θ = 30°) nanotubes, noticeably decreases for zigzag (θ = 0°) nanotubes and slightly decreases for the chiral family with θ = 19.1° (Figure 6a,b). The ratio EI/$D_{n}^{3}$ clearly increases for armchair (θ = 30°) and chiral (θ = 19.1°) nanotubes, and clearly decreases for zigzag (θ = 0°) nanotubes, for diameters $D_{n}$ < 1.5 nm (Figure 6c,d). That is, for SWBNNTs with diameters less than 1.5 nm, the ratios EA/$D_{n}$ and EI/$D_{n}^{3}$ decrease with $D_{n}$, when the chiral angle, θ, decreases from 30° (armchair) to 0° (zigzag). Although the ratio GJ/$D_{n}^{3}$ is nearly constant and equal to the value of the fitting parameter $\gamma_{\mathrm{BN}}$ (Equation (28)) for the SWBNNTs with diameter $D_{n}$ > 1.5 nm, similarly to what was observed for EA/$D_{n}$ and EI/$D_{n}^{3}$ ratios, the evolution of GJ/$D_{n}^{3}$ ratio with the decrease in nanotube diameter, $D_{n}$, is the opposite. For SWBNNTs with $D_{n}$ values less than 1.5 nm, GJ/$D_{n}^{3}$ increases for zigzag (θ = 0°) and decreases for chiral (θ = 19.1°) and armchair (θ = 30°) nanotubes. Consequently, for SWBNNTs with diameters less than 1.5 nm, the ratio GJ/$D_{n}^{3}$ decreases with the transition from zigzag to armchair structure, i.e., when the chiral angle, θ, increases from 0° to 30°. A comparable evolution for the ratio GJ/${{(D}_{n}{\;–\;D}_{0})}^{3}$ with the nanotube diameter was observed for the SWCNTs in a wide range of their chirality [47], with the difference that the value of GJ/${{(D}_{n}{\;–\;D}_{0})}^{3}$ becomes stable for SWCNTs with $D_{n}$ ≥ 1.0 nm.

**Figure 7:**
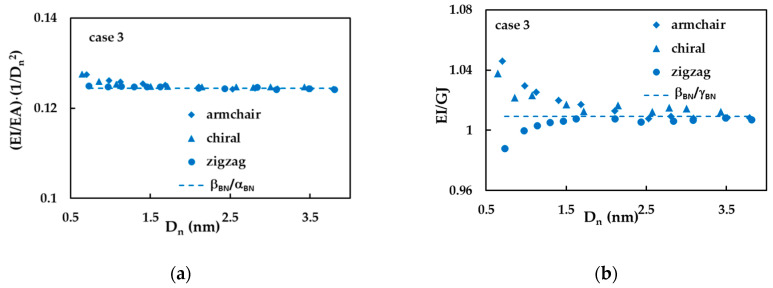
The evolutions of the ratios (**a**) (EI/EA)·(1/$D_{n}^{3}$) and (**b**) EI/GJ with the nanotube diameter, $D_{n}$, for case 3.

In order to clarify the evolutions of tensile and bending rigidities with the nanotube diameter and taking into account, Equation (22) for the nanotube mean diameter, the ratio (EI/EA)·(1/$D_{n}^{3}$) was considered. The evolution of the ratio (EI/EA)·(1/$D_{n}^{2}$) with $D_{n}$ is plotted in Figure 7a. The values of (EI/EA)·(1/$D_{n}^{2}$) are stable for high values of the nanotube diameter, $D_{n}$, and equal to the value of the ratio between fitting parameters $\beta_{\mathrm{BN}}/\alpha_{\mathrm{BN}}$, and slightly increases for armchair and chiral SWBBNTs for small nanotube diameters, $D_{n}$ < 1 nm. The evolution of the ratio between bending and torsional rigidities, EI/GJ, with $D_{n}$ is shown in Figure 7b. This ratio noticeably increases for armchair and chiral nanotubes and noticeably decreases for zigzag nanotubes, for diameters $D_{n}$ < 1.5 nm; for diameters greater than 1.5 nm tends to the value of the ratio $\beta_{\mathrm{BN}}/\gamma_{\mathrm{BN}}$.

**Figure 8:**
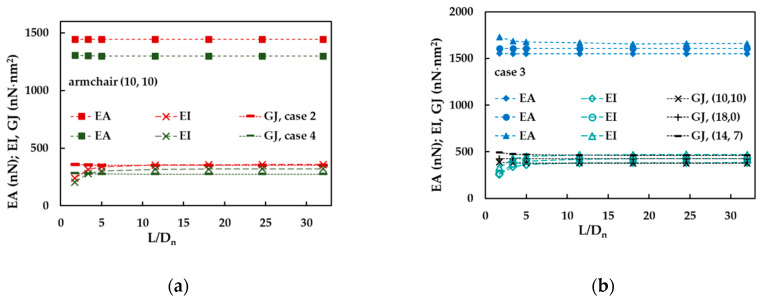
The evolutions of EA, EI and GJ rigidities with the aspect ratio, L/$D_{n}$, for: (**a**) (10, 10) armchair, cases 2 and 4, and (**b**) (10, 10) armchair, (18, 0) zigzag and (14, 7) chiral nanotubes, case 3.

It was also investigated the effect of the aspect ratio, L/$D_{n}$ (where L is the nanotube length), on the EA, EI and GJ rigidities of the SWBNNTs. Examples of tensile, bending and torsional rigidities as a function of L/$D_{n}$ are shown in Figure 8a,b. The tensile, EA, and torsional, GJ rigidities are almost constant in all range of nanotube aspect ratios for (10, 10) armchair and (18, 0) zigzag SWBNNTs, but slightly decrease for (14, 7) chiral nanotube, when L/$D_{n}$ is smaller than 5. The bending, EI, rigidity slightly increases for (10, 10) armchair, (18, 0) zigzag and (14, 7) chiral nanotubes when L/$D_{n}$ < 5, but above that, the value of EI is stable. In short, for aspect ratio values greater than 5, all rigidities are stable, and in some cases, even for values less than 5.

### 3.2. Elastic Moduli and Poisson’s Ratio of SWBNNTs

In this section, the Young’s and shear moduli and the Poisson’s ratio of SWBNNTs, calculated by Equations (23)–(25), respectively, are analysed. It is worth noting that Equation (23) together with relationships Equations (26) and (27) and the knowledge of the parameters $\alpha_{\mathrm{BN}}$, $\beta_{\mathrm{BN}}$ and $\gamma_{\mathrm{BN}}$ in Table 5 as well the nanotube diameter, $D_{n}$, and wall thickness, $t_{n}$, allow calculating Young’s modulus of the SWBNNTs without resorting to the numerical simulation as follows:

(29) $$E=\frac{\alpha_{\mathrm{BN}}\cdot D_{n}}{\pi \cdot t_{n}\cdot \sqrt{8\cdot \left(\frac{\beta_{\mathrm{BN}}}{\alpha_{\mathrm{BN}}}\right)\cdot D_{n}^{2}{\;–\;t}_{n}^{2}}}$$

Using the Equation (24) and relationships Equations (26)–(28), the shear modulus of the SWBNNTs can be calculated as follows:

(30) $$G=\frac{\gamma_{\mathrm{BN}}\cdot D_{n}}{2\cdot \pi \cdot \left(\frac{\beta_{\mathrm{BN}}}{\alpha_{\mathrm{BN}}}\right)\cdot t_{n}\cdot \sqrt{8\cdot \left(\frac{\beta_{\mathrm{BN}}}{\alpha_{\mathrm{BN}}}\right)\cdot D_{n}^{2}\;–t_{n}^{2}}}$$

Resorting to Equation (25) and relationships Equations (27) and (28), the Poisson’s ratio can be defined by an equation independent of the BNNTs diameter, as follows:

(31) $$\nu =\frac{\beta_{\mathrm{BN}}}{\gamma_{\mathrm{BN}}}\;–\;1$$

**Figure 9:**
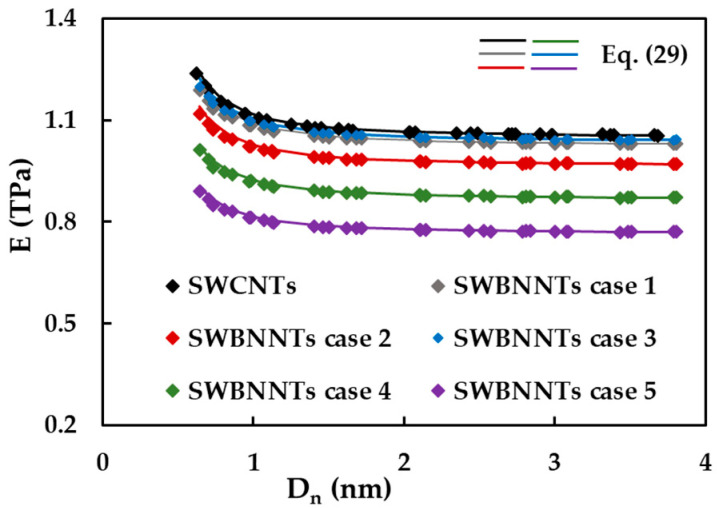
The evolution of the Young’s modulus, E, with the nanotube diameter, $D_{n}$, for SWBNNTs and SWCNTs.

Figure 9 shows the evolutions of Young’s modulus, E, with the nanotube diameter, $D_{n}$, for cases 1–5 of SWBNNTs and for SWCNTs. The E values calculated by Equation (29) are also plotted in Figure 9. Regardless of the case of the input parameters, chirality and the nature of the nanotube, whether non-carbon or carbon, Young’s modulus at the beginning decrease with the nanotube diameter, and then it becomes almost stable for the diameters $D_{n}$ > 1 nm. The mean value for which Young’s modulus of the cases 1 (E = 1.045 TPa) and 3 (E = 1.056 TPa) of SWBNNTs converges is slightly lower (by about 2%) than SWCNTs, which is 1.072 nm. The values for which Young’s modulus converges gradually decreases for the cases 2 to 4 and 5, E = 0.984, 0.884 and 0.781 TPa, respectively. It should be noted that Equation (29) allows calculating with sufficient accuracy Young’s modulus of SWBNNTs whatever the chirality and diameter without resorting to numerical simulation. Such a result was also reported for the case of SWCNTs in the previous study [50].

**Figure 10:**
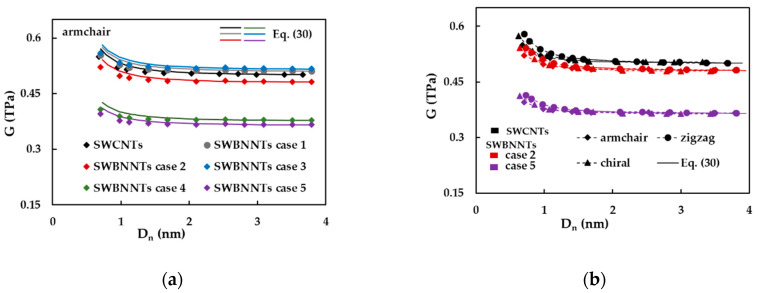
The evolutions of the shear modulus, G, as a function of the nanotube diameter, $D_{n}$, for: (**a**) armchair SWBNNTs (cases 1–5) and SWCNTs, and (**b**) cases 2 and 5 of armchair, zigzag and chiral SWBNNTs and SWCNTs.

Figure 10a shows the evolutions of the shear modulus, G, as a function of the nanotube diameter, $D_{n}$, for armchair nanotubes, considering cases 1–5 of SWBNNTs and SWCNTs. The G values calculated by Equation (30) are also plotted in Figure 10a. The shear modulus value of armchair nanotubes decreases with $D_{n}$, and, for high nanotube diameters, it becomes stable and tends to the value calculated by Equation (30). It can be noticed that Equation (30) does not allow calculating accurate values of the shear modulus of the armchair nanotubes with diameters $D_{n}$ < 1.5 nm. The highest converged average values of G assessed with Equation (31) are observed for the cases 1 (G = 0.516 TPa) and 3 (G = 0.522 TPa) of SWBNNTs, and the shear modulus of SWCNTs is about 1.7% lower than those in these cases. The value of the average shear modulus converges to 0.486, 0.382 and 0.369 TPa for cases 2, 4 and 5, respectively.

**Figure 11:**
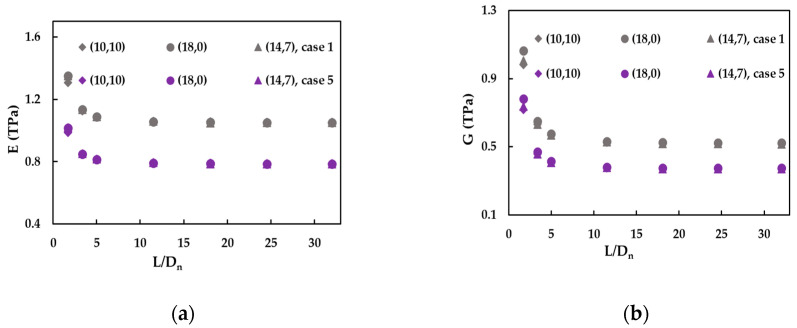
The evolutions of the (**a**) Young’s and (**b**) shear moduli, with the nanotube aspect ratio, L/$D_{n}$, for the cases 1 and 5 of (10, 10), (18, 0) and (14, 7) SWBNNTs.

In order to clarify the results shown in Figure 10a, the evolutions of the shear modulus, G, with nanotube diameter, $D_{n}$, were plotted in Figure 11b, for armchair, zigzag and θ = 19.1° family of chiral nanotubes, limiting to the cases 2 and 5 of SWBNNTs and the SWCNTs. Regarding the evolutions of G for three symmetry groups of nanotubes, the shear modulus is influenced by the chiral angle and slightly decreases from zigzag (θ = 0°) to armchair (θ = 30°) structures for SWBNNTs and SWCNTs with the diameters $D_{n}$ < 1.5 nm. With the increase in $D_{n}$, the shear modulus assumes identical almost stable values for armchair, zigzag and chiral nanotubes, which can be accurately described by Equation (30). This trend for the shear modulus evolution is in consonance with the results of Figure 6e,f, which show that the torsion rigidity, GJ, does not follow a linear relationship with the cubic power of the nanotube diameter, $D_{n}^{3}$, for diameters lower than 1.5 nm.

The results regarding the evolution of the E and G moduli with the aspect ratio, L/$D_{n}$, are shown in Figure 11a,b for armchair (10, 10), zigzag (18, 0) and chiral (14, 7) SWBNNTs. Both E and G moduli increase for the aspect ratio below 5 and stabilise for higher L/$D_{n}$ ratios. The Young’s modulus values obtained for (10, 10), (18, 0) and (14, 7) SWBNNTs are approximately equal whatever the aspect ratio, but the shear modulus values slightly decrease from zigzag to chiral and armchair SWBNNTs, when L/$D_{n}$ < 3.

**Figure 12:**
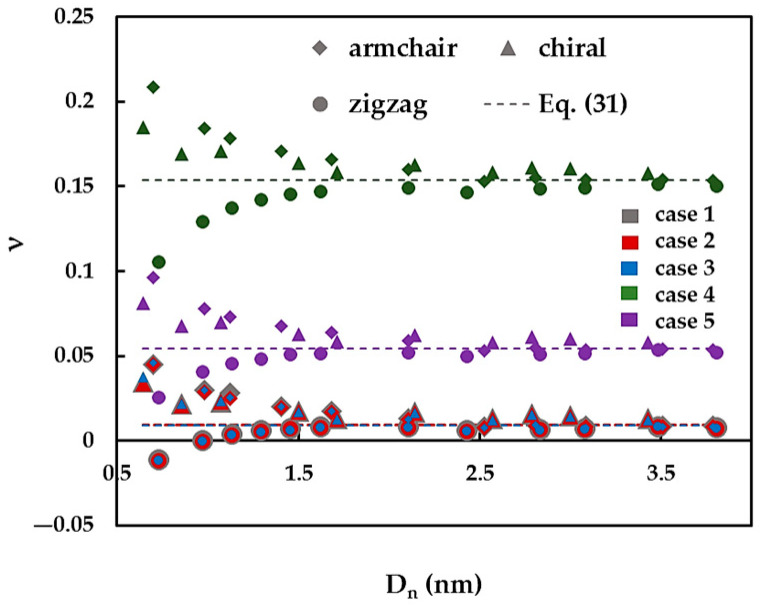
The evolution of the Poisson’s ration, ν, with the nanotube diameter, $D_{n}$, for SWBNNTs.

Figure 12 represents the evolution of the Poisson’s ratio, ν, calculated by Equation (25), with the nanotube diameter, $D_{n}$, for armchair, zigzag and θ = 19.1° family of chiral SWBNNTs. The ν values calculated by Equation (31), which does not depend on the $D_{n}$ values, are also indicated in Figure 12. For armchair, zigzag and chiral SWCNNTs with diameters $D_{n}$ > 1.5 nm, the Poisson’s ratio tends approximately to the value calculated by Equation (31). When the nanotube diameter decreases below 1.5 nm, the value of the Poisson’s ratio value increases, in the case of zigzag SWBNNTs, whereas for the cases of armchair and chiral SWBNNTs, the value of ν decreases. The Poisson’s ratio values calculated for cases 1–3 of the input parameters are approximately equal. Moreover, the SWBNNTs with small diameters, $D_{n}$ < 1 nm, show an auxetic behaviour (have a negative Poisson’s ratio). The mean value to which the Poisson’s ratio converges is about 0.15, 0.05 and 0.01 for the cases 4, 5 and 1–3, respectively.

It can be concluded that, for nanotube diameters $D_{n}$ < 1.5 nm, the Poisson’s ratio clearly depends on the chiral angle and increases from zigzag (θ = 0°) to armchair nanotubes (θ = 30°) SWCNNTs. This result agrees with those of Figure 7b, which shows that the ratio EI/GJ, between bending and torsion rigidities, does not have a constant value, for the nanotube diameter, $D_{n}$, below 1.5 nm.

Table 7 summarises the results of the current study on Young’s, E, and shear, G, moduli and Poisson’ ratio, ν, of SWBNNTs, calculated using several combinations of the bond length and force-field constants, which provided five sets of the input parameters for the numerical simulations. It is evident the considerable scattering for the E, G and ν values, caused by the variation of the input parameters in the framework of the same modelling approach (NCM/MSM) to describe the mechanical behaviour of SWBNNTs.

### 3.3. Comparison with Literature Results

Table 8 summarised the current results on the elastic property of SWBNNTs and also those from literature, including theoretical and experimental results.

To the best of our knowledge, the work of Arenal et al. [60] is the only study where Young’s modulus of individual SWBNNT was determined experimentally. Arenal et al. [60] used a high-resolution transmission electron microscope (HRTEM) and an atomic force microscope (AFM) set-up to carry out in situ uniaxial compression test and to obtain the force–displacement curve of an isolated SWBNNT and, consequently, the stress–strain curves considering three different values of nanotube wall thickness. Young’s modulus was calculated from the slope of these stress–strain curves in the linear regime. In two other experimental studies, Young’s modulus of multi-walled boron nitride nanotubes (MWBNNTs) was evaluated [61,62]. Chopra and Zettl [61] measured Young’s modulus from the thermal vibrational amplitude of a cantilevered MWBNNT examined in a transmission electron microscope (TEM), and Suryavanshi et al. [62] used the electric-field-induced resonance method inside TEM for this purpose.

The analytical and numerical results from the literature are related to the evaluation of the elastic properties of only non-chiral SWBNNTs with the exception of the work by Yan et al. [36], who reported shear modulus results for chiral SWBNNTs (see Table 8). Most of the works cited in Table 8 deal with the evaluation of Young’s modulus of SWBNNTs, and with regard to the evaluation of their shear modulus and Poisson’s ratio, the available results are considerably scarcer. It can be concluded from this table that the results of the current elastic property are in general in reasonably good agreement with those reported in the literature, including the experimental Young’s modulus values.

**Figure 13:**
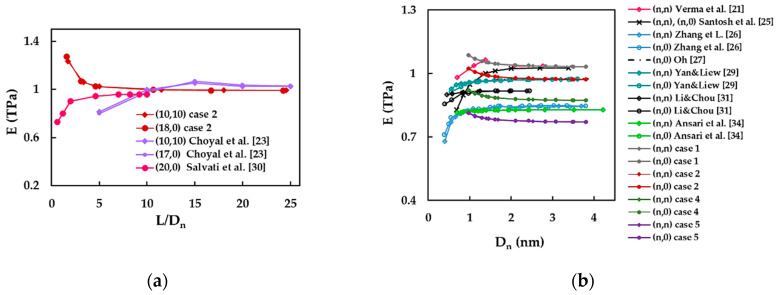
A comparison of the current results of Young’s modulus of SWBNNTs with those reported in the literature as a function: (**a**) the nanotube aspect ratio, L/$D_{n}$; (**b**) the nanotube diameter, $D_{n}$.

In order to simplify the comparison of the current results with those available in the literature, Young’s modulus was represented as a function of the nanotube aspect ratio, L/$D_{n}$, and the nanotube diameter, $D_{n}$, as shown in Figure 13a,b.

The current Young’s modulus evolutions as a function of the aspect ratio obtained for (10, 10) and (18,0) SWBNNTs, considering case 2 of the input parameters, are in satisfactory agreement with those reported by Choyal et al. [23] and Salvati et al. [30] for (10, 10) and (17, 0), and (20, 0) SWBNNTs, respectively, for L/$D_{n}$ > 8 (Figure 13a).

Regarding Young’s modulus evolution with the nanotube diameter, $D_{n}$, two trends have been reported in the literature: (i) Young’s modulus is almost constant over the range of SWBNNTs diameters [27,29,31,34]; and (ii) initially, Young’s modulus increases and then it becomes almost stable for high values of $D_{n}$ [21,25,26] (see Figure 13b). The current Young’s modulus results obtained for case 1 show a good agreement with the results of Verma et al. [21] for armchair SWBNNTs with the diameters $D_{n}$ ≥ 1.448 nm (difference of 0.77%), and Santosh et al. [25] for armchair and zigzag SWBNNTs with the diameters $D_{n}$ > 2.0 nm (difference of 0.93%). The Young’s modulus values calculated for case 2 are in particularly good consonance with those evaluated for SWBNNTs with high diameters $D_{n}$ > 2.0 nm by Yan and Liew [29] for armchair and zigzag SWBNNTs (difference of 0.37%), and by Oh [26] for zigzag SWBNNTs (difference of 0.28%). The Young’s modulus value reported by Li and Chou [31] is 4.64% higher than that obtained for case 4. The differences between Young’s modulus values obtained by Ansari et al. [34] and those calculated for cases 4 and 5 are 5.45% and 7.00%, respectively. For Young’s modulus values reported by Zhang et al. [26], the differences of 6.45% and 6.9% are observed when compared with cases 4 and 5, respectively.

**Figure 14:**
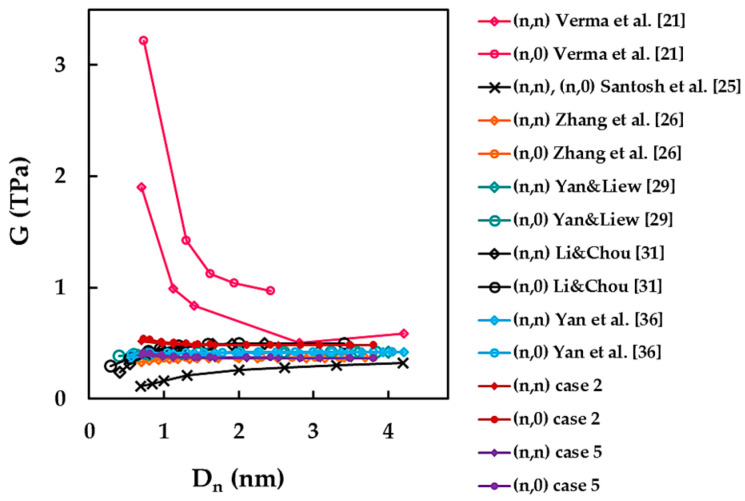
A comparison of the current shear modulus results with those reported in the literature as a function of the nanotube diameter, $D_{n}$.

Figure 14 shows the results of the current shear modulus obtained for cases 2 and 5 as a function of the SWBNNT diameter, D_n_, along with the results available in the literature (see also Table 8). Three trends were reported in the literature: (i) the shear modulus drastically decreases with nanotube diameter and then tends to stabilize when $D_{n}$ increases [21]; (ii) the shear modulus is almost constant through the whole range of nanotube diameters [26,29,36]; and (iii) in the beginning, the shear modulus slightly increases and then becomes almost stable for high values of $D_{n}$ [25,31]. When compared with current shear modulus results for case 2, a good agreement (difference of 1.77%) is observed for the results of Li and Chou [31] obtained for armchair and zigzag SWBNNTs with $D_{n}$ > 1.4 nm. Particularly good agreement (difference of 0.62%) is seen between shear modulus results for case 5 and those obtained by Zhang et al. [26] for armchair and zigzag SWBNNTs with $D_{n}$ > 1.6 nm.

Differences of 16.59% and 10.27% occur when the shear modulus values reported by Yan and Liew [29] are compared with those for cases 2 and 5, respectively. When comparing the shear modulus results of Yan et al. [36] with those for cases 2 and 5, the differences are 14.91% and 11.8%, respectively. Less agreement is noticed for the shear modulus results of Santosh et al. [25]. The difference between the values calculated by Santosh et al. [25] for SWBNNTs with $D_{n}$ > 1.3 nm and those obtained for case 5 is 16.83%. The biggest differences of at about 50% occur between the shear modulus values reported by Verma et al. [21] and the current results for case 2.

**Figure 15:**
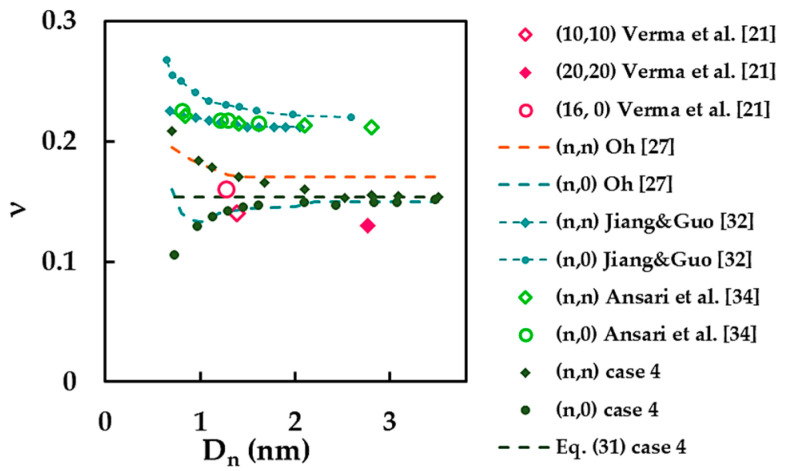
A comparison of the current Poisson’s ratio results with those from the literature as a function of the nanotube diameter, $D_{n}$.

Finally, the current results of the Poisson’s ratio are compared with the few available in the literature, as shown in Figure 15. The results obtained for case 4 were chosen as the only ones suitable for comparison. The ν value calculated by Equation (31) is also plotted in Figure 15.

The current Poisson’s ratio results show good agreement with the results of Oh [27] for armchair SWBNNTs in the range of their diameters from 0.983 nm to 1.684 nm and for zigzag SWBNNTs with $D_{n}$ ≥ 0.973 nm. The values of the Poisson’s ratio reported by Verma et al. [21] equal to 0.14 and 0.16 for (10, 10) and (16, 0) SWBNNTs, respectively, are satisfactorily close to the current results. Substantial differences of 39.18% and 44.95% were found when comparing the current results with those obtained by Jiang and Guo [32] and Ansari et al. [34], respectively.

In summary, the dissimilarity observed in between the current results and those available in the literature with regard to the determination of the elastic moduli and the Poisson’s ratio of SWBNNTs can be explained not only by different modelling and calculation approaches used to assess these properties but also by the variation of the input parameters within the same modelling and numerical simulation approach. In the current study, five sets of E, G and ν values were obtained, which permit finding a correspondence with the literature results for a broad selection of these. The Young’s and shear moduli values obtained in the numerical simulations, using the set of the input parameters corresponding to case 2, are in good agreement with a larger number of the Young’s and shear moduli results in the literature. Regarding Poisson’s ratio results, only case 4 of the input parameters leads to values comparable with those from the literature.

## 4. Conclusions

In this numerical simulation study, based on the NCM/MSM approach, a systematic evaluation of the elastic properties, including the bending, torsional and tensile rigidities, the shear and Young’s moduli and the Poisson’s ratio, of SWBNNTs was carried out over a wide range of chiral indices and nanotube diameters.

The main conclusions of the present study are as follows:

- Equations describing the relationship between each of the three rigidities and the nanotube diameter were obtained for SWBNNTs; five groups of the fitting parameters for the relationships Equations (26)–(28) were calculated, each for the corresponding input set used in the FE simulation;
- The relationships Equations (26)–(28) allow satisfactorily accurate analytical estimation of the Young’s and shear moduli and Poisson’s ratio of SWBNNTs, with the exception of the shear moduli and Poisson’s ratio of nanotubes with diameters lower than 1.5 nm;
- The variation of the input parameters for FE simulation leads to a considerable scatter of the calculated values of the SWBNNTs elastic properties; this allows selecting results that are in better agreement with those available in the literature and indicating the most appropriate set of input parameters for further numerical simulation studies.

## Figures and Tables

**Table 1:** Values of the B–N covalent bond length available in the literature.

Reference	Pokropivnyi [39]	Menon and Srivastava [40]	Kochaev [20]	Jiang and Guo [32]	Tapia et al. [41]
$a_{B-N}$ **, nm**	0.145	0.151	0.147	0.153	0.1447

**Table 2:** Bond force constants for BN nanostructures reported in the literature.

Reference	Method	k_r_, nN/nm	k_θ_, nN·nm/rad^2^	k_τ_, nN·nm/rad^2^
Rappé et al. [43]	UFF	676	2.358 ^1^	-
			1.122 ^2^	
Mayo et al. [44]	DREIDING	487	0.695	0.278
Li and Chou [31]		487	0.695	0.625
Jiang and Guo [32]	DFT	595	1.360 ^1^	-
			0.662 ^2^	
Genoese et al. [45]		585	1.347 ^1^	-
			0.641 ^2^	
Ansari et al. [33]		620	1.050	2.470
Tapia et al. [41]		617	0.627	0.132

^1^ For three-body angle of N–B–N. ^2^ For three-body angle of B–N–B.

**Table 3:** FE simulations input parameters of SWBNNTs and SWCNTs: beam element geometrical and mechanical properties.

Case		Reference	*l*, nm	Force Field Constants	d, nm	E_b_, GPa	G_b_, GPa	ν_b_
SWBNNTs	1	[33]	1.450	k_r_ = 620 nN/nm k_θ_ = 1.050 nN·nm/rad^2^ k_τ_ = 2.470 nN·nm/rad^2^	0.1645 Equation (13a)	4231 Equation (13b)	4976 Equation (13c)	0.21 Equation (15a)
	2	[45]	1.450	k_r_ = 585 nN/nm k_θ__1_ = 1.347 nN·nm/rad^2^ k_θ__2_ = 0.641 nN·nm/rad^2^ ^∗^k_τ_ = 2.470 nN·nm/rad^2^	0.1649 Equation (14a)	3973 Equation (14b)	4936 Equation (14c)	0.21 Equation (15b)
	3	[32]	1.530	k_r_ = 595 nN/nm k_θ__1_ = 1.360 nN·nm/rad^2^ k_θ__2_ = 0.662 nN·nm/rad^2^ ^∗^k_τ_ = 2.470 nN·nm/rad^2^	0.1649 Equation (14a)	4263 Equation (14b)	5208 Equation (14c)	0.24 Equation (15b)
	4	[41]	1.447	k_r_ = 617 nN/nm k_θ_ = 0.627 nN·nm/rad^2^ k_τ_ = 0.132 nN·nm/rad^2^	0.1275 Equation (13a)	6989 Equation (13b)	737 Equation (13c)	0.38 Equation (15a)
	5	[31]	1.450	k_r_ = 487 nN/nm k_θ_ = 0.695 nN·nm/rad^2^ k_τ_ = 0.625 nN·nm/rad^2^	0.1512 Equation (13a)	3930 Equation (13b)	1767 Equation (13c)	0.27 Equation (15a)
SWCNTs		[47] [48]	1.421	k_r_ = 652 nN/nm k_θ_ = 0.876 nN·nm/rad^2^ k_τ_ = 0.278 nN·nm/rad^2^	0.1470 Equation (13a)	5488 Equation (13b)	871 Equation (13c)	0.27 Equation (15a)

^∗^ Due to the lack of values reported, the k_τ_ force constant was calculated through k_τ_ = 24· D [49], where D is flexural rigidity, with D = 0.64 eV [33].

**Table 4:** Geometrical characteristics of the studied SWBNNTs and SWCNTs.

NT Symmetry Group	NT Type	(n, m)	θ°	SWBNNTs $D_{n},\;{\mathrm{nm}\;}^{1}$	SWCNTs, $D_{n},\;\mathrm{nm}$
non-chiral	armchair	(5, 5)	30	0.702	0.678
		(7, 7)		0.983	0.950
		(8, 8)		1.123	1.086
		(10, 10)		1.404	1.357
		(12, 12)		1.684	1.628
		(15, 15)		2.106	2.035
		(18, 18)		2.527	2.443
		(20, 20)		2.807	2.714
		(22, 22)		3.088	2.985
		(25, 25)		3.509	3.392
		(27,27)		3.790	3.664
	zigzag	(9, 0)	0	0.729	0.705
		(10, 0)		0.810	0.783
		(12, 0)		0.973	0.940
		(14, 0)		1.135	1.097
		(16, 0)		1.297	1.254
		(18, 0)		1.459	1.410
		(20, 0)		1.621	1.567
		(26, 0)		2.107	2.037
		(30, 0)		2.431	2.350
		(35, 0)		2.837	2.742
		(38, 0)		3.080	2.977
		(43, 0)		3.485	3.369
		(47, 0)		3.809	3.682
chiral	family 19.1°	(6, 3)	19.1	0.643	0.622
		(8, 4)		0.858	0.829
		(10, 5)		1.072	1.036
		(14, 7)		1.501	1.451
		(16, 8)		1.715	1.658
		(20, 10)		2.144	2.073
		(24, 12)		2.573	2.487
		(26, 13)		2.788	2.695
		(28, 14)		3.002	2.902
		(36, 12)		3.431	3.316
	n + m = 10	(6, 4)	23.4	0.707	0.683
		(7, 3)	17.0	0.720	0.696
		(8, 2)	10.9	0.743	0.718
		(9, 1)	5.2	0.773	0.747
	n + m = 20	(12, 8)	23.4	1.413	1.366
		(14, 6)	17.0	1.441	1.393
		(15, 5)	13.9	1.461	1.412
		(16, 4)	10.9	1.486	1.436
		(18, 2)	5.2	1.546	1.495
	n + m = 30	(16, 14)	27.8	2.107	2.037
		(18, 12)	23.4	2.120	2.049
		(21, 9)	17.0	2.161	2.089
		(22, 8)	14.9	2.181	2.108
		(24, 6)	10.9	2.228	2.154
		(25, 5)	8.9	2.256	2.181
		(27, 3)	5.2	2.319	2.242
		(28, 2)	3.4	2.355	2.276

^1^ Diameter, $D_{n}$, of SWBNNTs is calculated assuming B–N length $a_{B-N}$ = 0.147 nm defined by software Nanotube Modeler©.

**Table 5:** The fitting parameters $\alpha_{\mathrm{BN}}$, $\beta_{\mathrm{BN}}$ and $\gamma_{\mathrm{BN}}$ for SWBNNTs, considering the five cases of input values shown in Table 3.

Case	$\alpha_{\mathrm{BN}},\;\mathrm{nN}/\mathrm{nm}$	$\beta_{\mathrm{BN}},\;\mathrm{nN}/\mathrm{nm}$	$\gamma_{\mathrm{BN}},\;\mathrm{nN}/\mathrm{nm}$
1	1093.46	136.01	134.71
2	1029.90	128.11	126.95
3	1105.10	137.46	136.22
4	924.49	114.87	99.58
5	817.32	101.62	96.37

**Table 6:** The mean difference between the SWBNNTs rigidities values estimated with Equations (26)–(28) and the respective values obtained from FE analysis.

Case	Mean Difference, %		
	EA, nN	EI, nN·nm^2^	GJ, nN·nm^2^
1	0.21	0.62	0.19
2	0.20	0.62	0.18
3	0.21	0.64	0.19
4	0.32	0.76	0.38
5	0.24	0.66	0.24

**Table 7:** Young’s and shear moduli and Poisson’ ratio of SWBNNTs evaluated in the present numerical simulation study using different sets of the input parameters.

$\mathrm{Bond}\;\mathrm{Length},\;a_{B-N},\;\mathrm{nm}$	Force Field Constants			Elastic Properties *		
	k_r_, nN/nm	k_θ_, nN·nm/rad^2^	k_τ_, nN·nm/rad^2^	E, TPa	G, TPa	ν
1.450 [31,33,45]	487 [31]	0.695 [31]	0.625 [31]	0.781	0.369	0.05
	620 [33]	1.050 [33]	2.470 [33]	1.045	0.515	0.01
	585 [45]	1.347		0.984	0.486	0.01
		0.641 [45]				
1.530 [32]	595 [32]	1.360		1.056	0.522	0.01
		0.662 [32]				
1.447 [41]	617 [41]	0.627 [41]	0.132 [41]	0.884	0.382	0.15

* Converged mean values.

**Table 8:** A comparison of the current Young’s and shear moduli and the Poisson’s ratio results for boron nitride nanotubes with those reported in the literature.

Reference	Method	$t_{n},\;\mathrm{nm}$	Type of BNNT	E, TPa	G, TPa	ν	Comment
Hernandez et al. [3]	TBMD	0.340	(n, n)	0.894	-	0.26	average value
			(n, 0)	0.866	-	0.24	
Kochaev [20]	ab initio		(10, 10)	1.140	-	0.56	-
Santosh et al. [25]	MD: force–constant approach		(n, n); (n, 0)	1.017	0.326	-	converged average value
Verma et al. [21]	MD: TB potential	0.330	(n, n)	1.107	0.965	0.14	average value
			(n, 0)	1.044	1.555		
Choyal et al. [23]		0.340	(10, 10)	1.053	-	-	highest value for L/$D_{n}$ = 15
			(17, 0)	1.066			
Tao et al. [24]	MD: TB potential + FEM		(n, n)	0.911	-	-	converged average value
			(n, 0)	0.930			
Vijayaraghavan and Zhang [22]	MD: REBO	0.105	(10, 10)	2.8	-	-	-
Zhang et al. [26]	MD: DFTB	0.314	(n, n)	0.840	0.366	-	converged average value
			(n, 0)	0.844	0.368		
Oh [27]	CM: CL thermodynamic approach + TB potential	0.330	(n, n)	0.960	-	0.17	converged average value
			(n, 0)	0.975		0.15	
Yan and Liew [29]	NCM/MSM: representative cell	0.333	(n, n)	0.970	0.416	-	converged average value
			(n, 0)	0.967	0.418		
Yan et al. [36]	NCM/MSM: torsional vibrations		(n, n); (n, 0); (n, m)	-	0.418	-	converged average value
Jiang and Guo [32]	NCM/MSM: analytical solution	-	-	-	-	0.21	converged average value
						0.23	
Ansari et al. [34]		0.340	(n, n)	0.825	-	0.21	average value
			(n, 0)	0.823			
Salavati et al. [30]	NCM/MSM: beams		(n, n), (n, 0)	0.928	-	-	converged average value
Li and Chou [31]			(n, n)	0.916	0.465	-	converged average value
			(n, 0)	0.913	0.475		
Arenal et al. [60]	HRTEM-AFM + analytical	0.070	SWBNNT	1.11 ± 0.17	-	-	-
		0.090		0.87 ± 0.13			
		0.340		0.25 ± 0.04			
Chopra and Zettl [61]	TEM: thermal vibrational amplitude	-	MWBNNT	1.22 ± 0.24	-	-	-
Suryavanshi et al. [62]	TEM: electric-field-induced resonance	-	MWBNNT with $D_{n}$ from 34 to 94 nm	0.722 ± 0.14	-	-	average value for 18 MWBNNTs, E from 0.550 to 1.031 TPa
Current results	NCM/MSM: beams	0.340	(n, n); (n, 0); (n, m)	0.781	0.369	0.05	converged average value
				0.884	0.382	0.15	
				0.984	0.486	0.01	
				1.045	0.515		
				1.056	0.522		

## Data Availability

The data presented in this study are available on request from the corresponding author after obtaining permission of authorized person.

## Appendix A

**Table A1:** The number of elements and nodes of the FE meshes of SWBNNTs and SWCNTs.

(n, m)	SWBNNTs ^1^		SWCNTs ^1^	
	Number of Elements	Number of Nodes	Number of Elements	Number of Nodes
(5, 5)	5300	3540	5480	3660
(7, 7)	7420	4956	7672	5124
(8, 8)	8480	5664	8768	5856
(10, 10)	10,600	7080	10,960	7320
(12, 12)	12,720	8496	13,152	8784
(15, 15)	14,145	9450	16,305	10,890
(18, 18)	14,868	9936	19,566	13,068
(20, 20)	11,780	7880	21,740	14,520
(22, 22)	12,958	8668	23,914	15,972
(25, 25)	13,000	8700	27,175	18,150
(27,27)	14,040	9396	29,349	19,602
(9, 0)	5499	3672	5688	3798
(10, 0)	6110	4080	6320	4220
(12, 0)	7332	4896	7584	5064
(14, 0)	8554	5712	8848	5908
(16, 0)	9776	6528	10,112	6752
(18, 0)	10,998	7344	11,376	7596
(20, 0)	12,220	8160	12,640	8440
(26, 0)	14,092	9412	16,432	10,972
(30, 0)	14,190	9480	18,453	12,320
(35, 0)	20,020	13,370	23,396	15,618
(38, 0)	21,348	14,254	20,783	13,873
(43, 0)	24,455	16,324	27,577	18,404
(47, 0)	26,730	17,843	32,963	21,996
(6, 3)	4851	3240	5013	3348
(8, 4)	6468	4320	6684	4464
(10, 5)	8085	5400	8355	5580
(14, 7)	11,319	7560	11,697	7812
(16, 8)	12,936	8640	13,368	8928
(20, 10)	14,370	9600	16,860	11,260
(24, 12)	16,164	10,800	20,232	13,512
(26, 13)	17,004	11,348	23,010	15,366
(28, 14)	19,880	13,266	24,780	16,548
(36, 12)	26,901	17,948	31,008	20,704
(6, 4)	5330	3560	5510	3680
(7, 3)	5432	3628	5618	3752
(8, 2)	5600	3740	5798	3872
(9, 1)	5828	3892	6029	4026
(12, 8)	10,660	7120	11,020	7360
(14, 6)	10,864	7256	11,236	7504
(15, 5)	11,020	7360	11,395	7610
(16, 4)	11,200	7480	11,596	7744
(18, 2)	11,656	7784	12,058	8052
(16, 14)	14,130	9440	14,616	9764
(18, 12)	14,241	9507	13,101	8754
(21, 9)	14,478	9672	13,100	8754
(22, 8)	14,610	9760	13,224	8836
(24, 6)	14,984	10,001	13,534	9033
(25, 5)	14,286	9546	13,680	9140
(27, 3)	14,689	9814	14,055	9390
(28, 2)	14,906	9959	14,267	9532

^1^ The length of the SWBNNTs and SWCNTs was about 30× greater than the nanotube diameter, $D_{n}$.
